# COVID-19 Mortality in Europe, by Latitude and Obesity Status: A Geo-Spatial Analysis in 40 Countries

**DOI:** 10.3390/nu14030471

**Published:** 2022-01-21

**Authors:** Stefanos Tyrovolas, Thomas Tsiampalis, Marianthi Morena, Angela Y. M. Leung, Antigoni Faka, Christos Chalkias, Sotirios Tsiodras, Dimosthenes Panagiotakos

**Affiliations:** 1 School of Health Science and Education, Harokopio University, 17671 Athens, Greece; stefanos.tyrovolas@polyu.edu.hk (S.T.); ttsiam@hua.gr (T.T.); 2 Research, Innovation and Teaching Unit, Parc Sanitari Sant Joan de Déu, 08830 Sant Boi de Llobregat, Spain; 3 Instituto de Salud Carlos III, Centro de Investigación Biomédica en Red de Salud Mental, CIBERSAM, 28029 Madrid, Spain; 4 School of Nursing, The Hong Kong Polytechnic University, Hung Hom, Kowloon GH506, Hong Kong; angela.ym.leung@polyu.edu.hk; 5 School of Environment, Geography, and Applied Economics, Harokopio University, 17671 Athens, Greece; marianthi.morena@gmail.com (M.M.); afaka@hua.gr (A.F.); xalkias@hua.gr (C.C.); 6 School of Medicine, National and Kapodistrian University of Athens, 11527 Athens, Greece; sotirios.tsiodras@gmail.com

**Keywords:** COVID-19, incidence, fatality, obesity, sunlight exposure, Vitamin D synthesis

## Abstract

On 30 January 2020, the World Health Organization (WHO) declared the current novel coronavirus disease 2019 (COVID-19) as a public health emergency of international concern and later characterized it as a pandemic. New data show that excess body mass and vitamin D deficiency might be related to the disease severity and mortality. The aim of this study was to evaluate whether latitude, as a proxy of sunlight exposure and Vitamin D synthesis, and prevalent obesity among European populations, is related to COVID-19 spread and severity. European COVID-19 data (incidence and fatality), including information on the prevalence of obesity, social distancing, and others were obtained by the “Our World in Data” website on 17 April 2021. Adjusted analysis showed that higher COVID-19 incidence and fatality were pictured in countries being in higher latitude, both during the whole period, as well as, during the time period 1 November 2020–31 March 2021. Higher incidence and fatality of COVID-19 were observed where the prevalence of overweight/obesity was higher during the whole time period, whereas during the time period 1 November 2020–31 March 2021, only COVID-19 incidence was higher but not a fatality. The present results provide insights for targeted interventions and preventive strategies against COVID-19.

## 1. Introduction

On 30 January 2020, the current novel coronavirus disease 2019 (COVID-19) was declared an emergency of public health and international concern by the World Health Organization (WHO), and a few days later, a pandemic [1,2]. On 31 December 2019, the first cases of unknown aetiology pneumonia were reported from Wuhan City, China [3]. After this date, extended international transmission outside China, was observed among various European Union (EU) countries (e.g., France, Germany) and globally [4,5].

Large global and European geospatial variations have been reported thereafter [6,7]. Dye et al. [8] reported that COVID-19 mortality among European countries, marking as final date 31 July, varied more than 100-fold. These geospatial variations are mainly attributed to the implementation efficacy and timing of several prevention measures, including social distancing and other health policies to prevent and contain the spread of COVID-19 [6]. Other determinants, such as population density, social relationships divergency at local scales as well as mobility patterns have been related to COVID-19 mortality geospatial variations [9]. Moreover, other factors, including age, gender, ethnicity, social settings, and accumulated comorbidities, seem to be associated with an increased risk in disease severity and mortality [10].

Among the various comorbidities and disease entities related to increased COVID-19 severity, obesity has been characterized as an emerging COVID-19 risk factor, especially among industrialized countries [11]. A strong association between obesity and previous epidemics such as SARS and MERS has been previously reported [12]. Recent meta-analyses report that overweight and obese COVID-19 patients are severely affected by COVID-19 [13] andCOVID-19 associated hospitalization is higher among obese individuals [14]. Underlying this association, there may be obesity related adverse effects on the immune system and chronic inflammation [15].

While obesity seems to be related to immune system defects, vitamin D is well known to be a vital regulator for the immune system [16]. Vitamin D can be obtained from nutrition sources but mainly is produced after exposure to UV B-light, and as it is expected its synthesis is affected by latitude of residence and seasonality effects [17]. Low vitamin D status is a well-known European public health problem and has been related to various infectious and chronic diseases [18]. Until today a limited amount of data regarding COVID-19 severity and vitamin D status exists and the link between vitamin D levels and COVID-19 health outcomes remain controversial. A recent meta-analysis showed that an individual with insufficient vitamin D levels was 80% more likely to acquire COVID-19 infection compared to a counterpart with normal vitamin D levels [17]. However, original studies analysing individual data from a variety of countries, showed conflicting results [18].

Even though the scientific interest regarding obesity, vitamin D, and COVID-19 spread, and mortality has increased, there is a lack of evidence from cross-national epidemiologic data. A few original cohort studies from various settings do exist [19,20], however, studies on the epidemiology of obesity and vitamin D levels, especially from European settings, are limited. For this reason, the aim of this study was to evaluate the hypothesis of whether latitude, a proxy of sunlight exposure, and prevalent obesity among European populations, plays a role in COVID-19 spread and severity.

## 2. Materials and Methods

### 2.1. Epidemiological Data Sources

#### 2.1.1. Incidence, and Fatality of COVID-19 in Europe

All the epidemiological data for COVID-19 in the European region, namely its incidence and fatality, were provided by the “Our World in Data” website on 17 April 2021. Incidence of COVID-19 was defined as the number of new cases and fatality of COVID-19 was defined as the number of deaths reported in each European country. In order to account for differences in the populations among the European countries, both the incidence and the fatality of COVID-19 were expressed as the number of new cases and deaths per 1 million population, respectively.

#### 2.1.2. Prevalence of Overweight/Obesity in Europe

The latest data on the prevalence of overweight/obesity during 2016 in each European country, were provided by the “Our World in Data” website [21], which are based on the latest statistics published by the World Health Organization (WHO). Overweight was defined as Body Mass Index (BMI) equal to or greater than 25 kg/m^2^, while obesity was defined as BMI over 30 kg/m^2^. Finally, based on the median of the prevalence of overweight/obesity, European countries were divided into two categories: (i) Overweight/Obesity ≤ 62.3% and (ii) Overweight/Obesity > 62.3%.

#### 2.1.3. Government Response to the COVID-19 Pandemic, Stringency Index

Data on the stringency index in each European country were provided by the “Our World in Data” website on 17 April 2021 [21]. The specific index constitutes a composite measure representing the government response to the COVID-19 pandemic. More specifically, the stringency index is an additive score of nine indicators, which are being measured on an ordinal scale, and then they are rescaled to vary from 0 to 100. The indicators being used for the calculation of the final index are presented in Table 1.

### 2.2. Spatial Data

#### Latitude

Latitude data were provided by https://developers.google.com/public-data/docs/canonical/countries_csv (accessed April 2021) using each country’s capital city geographical coordinates.

### 2.3. Statistical Analysis

Choropleth maps were created in order to visualize the spatial distribution of COVID-19 incidence and fatality, expressed as the number of new cases and deaths per 1 million population, respectively, by using the quantile classification method. The specific method generates comparable classification schemes, considered to be extremely useful when there is a need to observe relative positions and make comparisons across maps. Incidence Rate Ratios (IRR) and their corresponding 95% Confidence Interval (CI) were evaluated through univariable and multivariable Poisson regression (containing the median population’s age—in years, the country’s Stringency index—in continuous form, the population density—expressed as population per km^2^ and the GDP per capita), that was used to investigate the association between the countries’ latitude and the COVID-19 incidence and fatality, both in the whole time period (1 January 2020–17 April 2021), as well as during the time period 1 November 2020–31 March 2021 (the latter representing the “winter seasonality” period). Subgroup analysis was also performed based on the prevalence of overweight/obesity, aiming at investigating if the examined association between the countries’ latitude and the COVID-19 incidence and fatality was being significantly differentiated according to the level of overweight/obesity. All statistical analyses were performed in STATA version 17 (STATA Corp Ltd., College Station, TX, USA), and statistical significance was set at *p*-value < 0.05.

## 3. Results

**Figure 1:**
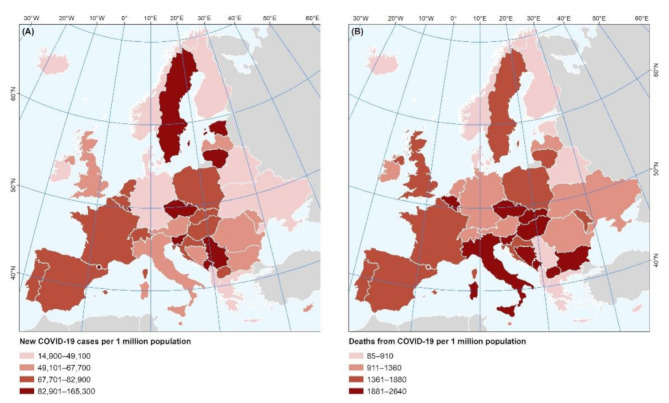
Spatial variability of the COVID-19 (**A**) incidence and (**B**) fatality during the time period 1 January 2020–17 April 2021.

As depicted in Figure 1, there were at least 14,989 new COVID-19 cases and at least 85 deaths from COVID-19 per 1 million population during the time period 1 January 2020–17 April 2021, with the lowest incidence being presented in Finland (14,989 cases/1 million) and the lowest fatality rates presented in Iceland (85 deaths/1 million), while the highest incidence was presented in Andorra (165,288 cases/1 million) and the highest fatality rates were presented in the Czech Republic (2640/1 million).

In addition, after adjusting for the median population’s age, stringency index, population density, and GDP per capita (Table 2), a significantly higher COVID-19 incidence and fatality were observed in European countries being in higher latitude, both during the whole time period [IRR (95% CI): Incidence = 1.07037 (1.07032, 1.07056), Fatality = 1.08664 (1.08618, 1.08699)], as well as, during the time period 1 November 2020–31 March 2021 [IRR (95% CI): Incidence = 1.07581 (1.07560, 1.07592), Fatality = 1.09222 (1.09148, 1.09289)]. Furthermore, as depicted in Table 2, the relationship between the countries’ latitude and the COVID-19 incidence and fatality, was significantly stronger in the European countries where the prevalence of overweight/obesity was higher.

**Figure 2:**
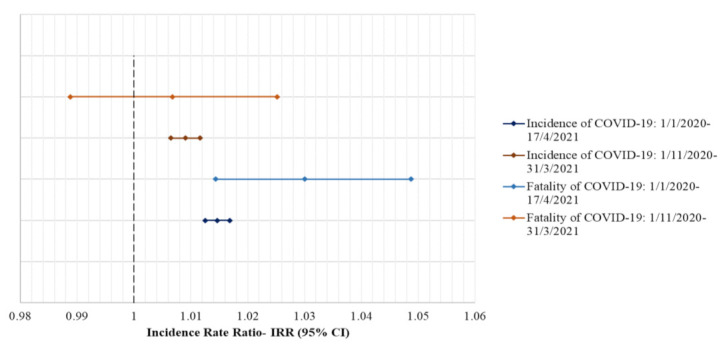
Results from Poisson regression analysis regarding the relationship of the prevalence of overweight/obesity with the incidence and fatality of COVID-19 per 1 million population during the whole time period (1 January 2020–17 April 2021) and during the time period 1 November 2020–31 March 2021; Notes: Results are presented in the form: IRR (95% CI); IRR: Incidence Rate Ratio; CI: Confidence Interval; IRRs are presented per 1% increment in the prevalence of overweight/obesity, and they are adjusted for the median population’s age, the stringency index, the population density and the GDP per capita; Stringency Index: A composite measure based on nine response indicators including school closures, workplace closures, and travel bans, rescaled to a value from 0 to 100 (100 = strictest).

As for the relationship between the COVID-19 incidence and fatality with the prevalence of overweight/obesity, after adjusting for the median population’s age and stringency index, (Figure 2), a significantly higher incidence and fatality of COVID-19 were observed in the European countries where the prevalence of overweight/obesity was higher, during the whole time period [IRR (95% CI): Incidence = 1.01463 (1.01251, 1.01681), Fatality = 1.03000 (1.01438, 1.04587)], whereas during the time period 1 November 2020–31 March 2021, only COVID-19 incidence rates were higher but not fatality rates [IRR (95% CI): Incidence = 1.00903 (1.00652, 1.01160), Fatality = 1.00677 (0.98875, 1.02515)].

## 4. Discussion

The present study analyzed the relation of obesity and vitamin D status (using the geographical latitude proxy) with COIVD-19 incidence and mortality among 40 European countries from the beginning of the pandemic to 17 April 2021. First, an unadjusted analysis of the current COVID-19 data showed a high variability of COVID-19 incidence and mortality among European countries. Second, adjusted analyses depicted higher COVID-19 incidence and fatality rates in European countries of higher latitude. Third, a higher prevalence of excess body mass was related to higher incidence and fatality rates of COVID-19 among populations living in European countries. Currently, there is limited information about the relation of high obesity/overweight rates as well as of low vitamin D levels (as expressed through the variation of high and low latitudes) and COVID-19 spread and mortality, specifically among European residents; the analysis presented herein could be used to inform public health authorities in strategic planning and preventive efforts against the COVID-19 pandemic, in specific population groups of countries with higher latitude.

The role of excess body mass on COVID-19 severity has been a matter of intense research. Our epidemiological analysis based on population data from 40 European countries, shows that obesity is associated with higher COVID-19 spread and mortality. Our results are in line with previous studies analysing individual data around the world [20]. In addition, new insights from meta-analyses support our findings [13]. The pathophysiologic mechanism supporting this relation is based on the role of the immune system and obesity-induced adipose tissue inflammation. Specifically, abnormal body mass has been related to excess secretion of various inflammatory markers such as those of the interleukin family [22]. These inflammatory markers are also implicated in severe COVID-19 pathophysiology [23]. Apart from inflammation, obesity has been associated with immune cell deficiency, such as the mucosal associated invariant T (MAIT) cells, that may have a crucial role in the defence mechanism against the new virus [24]. Our finding and the potential link of obesity with adverse effects on the immune system may serve as an important argument in favor of specific COVID-19 preventive and intervention strategies for the overweight and obese population of Europe.

Levels of vitamin D in the human body may have important enhancing effects on the immune system and its performance [16]. Our epidemiologic analysis in order to capture insufficient populations’ vitamin D levels used the geographic latitude as a proxy variable [25]. Our data reported that among the countries at higher latitude (with expected less sun and UV exposure [26,27]), the COVID-19 spread and mortality was higher. Our analysis on geographic latitude/Vitamin D and mortality rates is also supported by previous studies in the field, where sufficient sunlight and daily UV dose has been linked with lower COVID-19 population deaths [28]. Our results are also in line with new studies in the field and are also supported by recent meta-analyses [18,29]. However, a recent study from the UK reported no relation between 25(OH)D concentration and the severity of COVID-19 infection and fatality [30]. In addition to that, Chen et al. reported recently that vitamin D supplementations are not improving COVID-19 clinical outcomes among patients [31]. The potential pathophysiological mechanism supporting the relation between vitamin D and COVID-19 severity symptoms and mortality is centered on vitamins D immunomodulant, anti-inflammatory, and also anti-infective potential roles [32]. One proposed mechanism involves the local lung milieu and potential anti-inflammatory effects of vitamin D. It is well known that lung cells convert the 25(OH)D, which is the inactive form of vitamin D, to its active compound the 1,25(OH) vitamin D, reducing proinflammatory cytokines but also promoting the production of cathelicidin an epithelial peptide with the potential of antiviral activity [33]. Along with the aforementioned, it should be taken into account that vitamin D deficiency has been related to obesity, while European data are marking high population obesity [34]. Although the exact pathway of vitamin D deficiency among obese individuals is unknown, low UV exposure and increased metabolic clearance have been proposed as possible explanations [35]. Our results taking into account that in Europe, 40% of the population is vitamin D deficient [25], in parallel with the high obesity rates, could provide further evidence for the enhancement of preventing strategies against vitamin D deficiency; such strategies may be associated with a potential benefit in COVID-19 outcomes.

### Limitations

This is among the first studies of this kind using available COVID-19 mortality data and analyzing their relation to prevalent obesity and regional latitude, among European regions. However, this study shares common limitations as previous studies with similar methodology reports [6]. There are challenges in capturing uncertainty regarding COVID-19 data, government social distancing measures and their implementation, diversities in surveillance definitions among different locations, which may not fully capture temporal trends of COVID-19 mortality and disease severity. Variations in mortality rates among countries depend on several epidemiological indices like the quality of the surveillance system, testing capacities (significant under-reporting of deaths has been recently described), the demographics of the populations, and the burdening of the health system [36,37]. To make our comparisons among countries robust our analysis included factors such as median population’s age, the stringency index, the population density, and the GDP per capita. Furthermore, our study couldn’t adjust for other mediating factors related to vitamin D obtainment such as dietary habits, use of sunblock, and skin pigmentation. It has been reported that obesity is related to vitamin D deficiency [35], through volumetric dilution of vitamin D into the greater volumes of fat, serum, liver, and muscle. This is a fact that may have influenced our analysis. This issue is of course also linked to a volumetric dilution of vitamin D into the greater volumes of fat, serum, liver, and muscle present in obese people. Finally, the potential interaction of high latitude with other factors like urban living and ethnicity may confound the association of latitude with COVID19 fatality.

## 5. Conclusions

Herein we report that among 40 European countries, excess body mass and Vitamin D deficiency (expressed through each country’s latitude) were related to increased COVID-19 incidence and mortality. Obesity has deleterious effects on a person’s mental as well as physical health. Our findings give insights that may help clinicians and public health stakeholders to plan targeted nutrition related interventions and overweight/obesity related prevention policies as potential supportive strategies against COVID-19 spread and related outcomes. In addition, similar projects could serve as a guide for adopting the best preventive strategies for future outbreaks of similar magnitude.

## Figures and Tables

**Table 1:** Description of the 13 indicators of which the stringency index consists.

Indicators of Stringency Index	Scoring-Description
School closures	0—No measures, 1—recommend closing, 2—Require closing (only some levels or categories, e.g., just high school, or just public schools), 3—Require closing all levels
Workplace closures	0—No measures, 1—recommend closing (or work from home), 2—require closing (or work from home) for some sectors or categories of workers, 3—require closing (or work from home) all but essential workplaces (eg grocery stores, doctors)
Cancel public events	0- No measures, 1—Recommend cancelling, 2—Require cancelling
Restrictions on gatherings	0—No restrictions, 1—Restrictions on very large gatherings (the limit is above 1000 people), 2—Restrictions on gatherings between 100–1000 people, 3—Restrictions on gatherings between 10–100 people, 4—Restrictions on gatherings of less than 10 people,
Close public transport	0—No measures, 1—Recommend closing (or significantly reduce volume/route/means of transport available), 2—Require closing (or prohibit most citizens from using it)
Public information campaigns	0—No COVID-19 public information campaign, 1—public officials urging caution about COVID-19, 2—coordinated public information campaign (e.g., across traditional and social media)
Stay at home	0—No measures, 1—recommend not leaving house, 2—require not leaving house with exceptions for daily exercise, grocery shopping, and ‘essential’ trips, 3—Require not leaving house with minimal exceptions (e.g., allowed to leave only once every few days, or only one person can leave at a time, etc.)
Restrictions on internal movement	0—No measures, 1—Recommend movement restriction, 2—Restrict movement,
International travel controls	0—No measures, 1—Screening, 2—Quarantine arrivals from high-risk regions, 3—Ban on high-risk regions, 4—Total border closure
Testing policy	0—No testing policy, 1—Only those who both (a) have symptoms AND (b) meet specific criteria (e.g., key workers, admitted to hospital, came into contact with a known case, returned from overseas), 2—testing of anyone showing COVID-19 symptoms, 3—open public testing (e.g., “drive through” testing available to asymptomatic people)
Contract tracing	0—No contact tracing, 1—Limited contact tracing—not done for all cases, 2—Comprehensive contact tracing—done for all cases
Face coverings	0—No policy, 1—Recommended, 2—Required in some specified shared/public spaces outside the home with other people present, or some situations when social distancing not possible, 3—Required in all shared/public spaces outside the home with other people present or all situations when social distancing not possible, 4—Required outside the home at all times regardless of location or presence of other people
Vaccination policy	0—No availability, 1—Availability for ONE of following: key workers/clinically vulnerable groups/elderly groups, 2—Availability for TWO of following: key workers/clinically vulnerable groups/elderly groups, 3—Availability for ALL of following: key workers/clinically vulnerable groups/elderly groups, 4—Availability for all three plus partial additional availability (select broad groups/ages), 5—Universal availability

**Table 2:** Results from Poisson regression analysis regarding the relationship of the countries’ latitude with the incidence and fatality of COVID-19 per 1 million population during the whole time period (1 January 2020–17 April 2021) and during the time period 1 January 2020–31 March 2021, both in the total sample, as well as according to the prevalence of overweight/obesity.

IRR (95% CI)		Incidence of COVID-19 per 1 Million Population		Fatality of COVID-19 per 1 Million Population		Adjusted for:
		Whole Time Period (1 January 2020–17 April 2021)	Time Period of Interest (1 November 2020–31 March 2021)	Whole Time Period (1 January 2020–17 April 2021)	Time Period of Interest (1 November 2020–31 March 2021)	
**Total sample**	**Model 1**	1.01275 (1.01271, 1.01279)	1.01194 (1.01189, 1.01199)	1.0091 (1.0088, 1.0094)	1.01202 (1.01169, 1.01235)	Univariable
	**Model 2**	1.07037 (1.07032, 1.07056)	1.07581 (1.07560, 1.07592)	1.08664 (1.08618, 1.08699)	1.09222 (1.09148, 1.09289)	Median population’s age, Stringency index, Population density, GDP per capita
**Overweight/Obesity ≤ 62.3%**	**Model 1**	1.00212 (1.00206, 1.00218)	1.00194 (1.00188, 1.00201)	1.00498 (1.00461, 1.00534)	1.00243 (1.00199, 1.00288)	Univariable
	**Model 2**	1.02637 (1.02616, 1.02648)	1.03833 (1.03811, 1.03848)	1.04060 (1.04001, 1.04122)	1.05597 (1.05519, 1.05733)	Median population’s age, Stringency index, Population density, GDP per capita
**Overweight/Obesity > 62.3%**	**Model 1**	1.05132 (1.05126, 1.05139)	1.04314 (1.04306, 1.04323)	1.05303 (1.05255, 1.05352)	1.04673 (1.04615, 1.04731)	Univariable
	**Model 2**	1.18022 (1.17975, 1.18048)	1.16453 (1.16422, 1.16488)	1.19495 (1.19371, 1.19623)	1.17938 (1.17768, 1.18232)	Median population’s age, Stringency index, Population density, GDP per capita

**Notes**: Results are presented in the form: IRR (95% CI); IRR: Incidence Rate Ratio; CI: Confidence Interval; IRRs are presented per 1^o^ increment in the latitude; Stringency Index: A composite measure based on nine response indicators including school closures, workplace closures, and travel bans, rescaled to a value from 0 to 100 (100 = strictest).

## Data Availability

Data used in the present work are freely available in the https://ourworldindata.org/ accessed on 1 April 2021.
